# Supplementary material for: The Effect of the Yingyangbao Complementary Food Supplement on the Nutritional Status of Infants and Children: A Systematic Review and Meta-Analysis
Source: Nutrients. 2019 Oct 8;11(10):2404. doi: 10.3390/nu11102404 (PMC6835467; doi:10.3390/nu11102404)
Supplement: Supplementary file 1 [file nutrients-11-02404-s001.pdf]

Supplementary Materials

Table of Contents

Table S1: Risk of bias of post-only with concurrent control studies assessed with Methodological Index for Non-randomized Studies (MINORS)...3
Table S2: Risk of bias of pre-post studies assessed with Methodological Index for Non-randomized Studies (MINORS)..4
Table S3: Composition of three types of YYB..5
Table S4: Effects of YYB by intervention duration on hemoglobin concentration (g/L), height (cm), weight (kg) and Z-scores. 6
Table S5: Effects of YYB by intervention duration on prevalence of anemia, stunting, underweight and wasting...8
Table S6: Effects of YYB on hemoglobin concentration (g/L), height (cm), weight (kg) and Z-scores among studies of high and medium quality[1]..10
Table S7: Effects of YYB on prevalence of anemia, stunting, underweight and wasting among studies of high and medium quality[1]..11
Figure S1: Effects of YYB on hemoglobin level (g/L) among post-only with concurrent control studies (a) and pre-post studies (b) using mean difference as the effect measure. 12
Figure S2: Effects of YYB on height (cm) among post-only with concurrent control studies (a) and pre-post studies (b) using mean difference as the effect measure. 13
Figure S3: Effects of YYB on weight (kg) among post-only with concurrent control studies (a) and pre-post studies (b) using mean difference as the effect measure. 14
Figure S4: Effects of YYB on height-for-age z score (HAZ) among post-only with concurrent control studies (a) and pre-post studies (b) using mean difference as the effect measure........15
Figure S5: Effects of YYB on weight-for-height z score (WHZ) among post-only with concurrent control studies (a) and pre-post studies (b) using mean difference as the effect measure........16
Figure S6: Effects of YYB on weight-for-age z score (WAZ) among post-only with concurrent control studies (a) and pre-post studies (b) using mean difference as the effect measure........17
Figure S7: Effects of YYB on anemia prevalence among post-only with concurrent control studies (a) and pre-post studies (b) using risk ratio as the effect measure.................................18
Figure S8: Effects of YYB on stunting prevalence among post-only with concurrent control studies (a) and pre-post studies (b) using risk ratio as the effect measure.................................19
Figure S9: Effects of YYB on underweight prevalence among post-only with concurrent control studies (a) and pre-post studies (b) using risk ratio as the effect measure.................................20
Figure S10: Effects of YYB on wasting prevalence among post-only with concurrent control studies (a) and pre-post studies (b) using risk ratio as the effect measure.................................21
Figure S11: Effects of YYB on anemia prevalence among post-only with concurrent control

studies (a) and pre-post studies (b) using risk difference as the effect measure. 22
Figure S12: Effects of YYB on stunting prevalence among post-only with concurrent control studies (a) and pre-post studies (b) using risk difference as the effect measure. 23
Figure S13: Effects of YYB on underweight prevalence among post-only with concurrent control studies (a) and pre-post studies (b) using risk difference as the effect measure. 24
Figure S14: Effects of YYB on wasting prevalence among post-only with concurrent control studies (a) and pre-post studies (b) using risk difference as the effect measure. 25
Figure S15: Funnel plot of effects of YYB on anemia prevalence among post-only with concurrent control studies. 26
Figure S16: Funnel plot of effects of YYB on anemia prevalence among pre-post studies. 27

Table S1: Risk of bias of post-only with concurrent control studies assessed with Methodological Index for Non-randomized Studies (MINORS).

First Author	Year	Clearly stated aim	Consecutive inclusion	Prospective collection of data	Appropriate endpoints	Unbiased assessment of study endpoint	Appropriate follow-up period	Loss to follow up <5%	Calculation of study size	Control group with gold standard	Contemporary groups	Baseline equivalence of groups	Adapted statistical analyses	SUM	Overall quality[a]
Shuai Li	2017	7	6	7	7	3	5	6	2	4	7	7	5	66	High
Qin Hu	2016	7	3	7	5	3	5	4	2	3	7	7	4	57	High
Xiaoting Ding	2016	7	3	6	6	3	4	5	2	4	7	4	7	58	High
Yanfeng Zhang	2016	7	5	6	6	4	6	3	3	4	7	4	4	59	High
Lingyun Ren	2014	7	2	6	5	2	5	5	2	2	7	3	4	50	Medium
Shangming Li	2014	7	2	6	4	2	4	4	2	4	7	3	3	48	Low
Songli Fan	2013	7	4	7	5	3	2	3	2	3	7	3	3	49	Low
Wenhao Li	2013	7	6	7	7	3	2	6	2	4	7	7	6	64	High
Wenli Zhao	2012	6	3	6	5	3	4	3	3	4	7	4	5	53	Medium
Zhifeng Fang	2010	6	2	6	5	3	3	3	2	3	7	4	4	48	Low
Yuying Wang	2009	7	3	7	5	3	4	3	2	2	7	4	5	52	Medium
Yuying Wang	2004	7	3	7	5	2	4	4	2	2	7	4	4	51	Medium
Shuhua Ni	1995	7	2	7	5	2	3	3	1	4	7	3	4	48	Low

The overall quality is based on total MINORS score. In post-only studies, we defined a paper with a score higher than 53 a high-quality one, 50-53 a moderate-quality one, and lower than 50 a low-quality one.

Table S2: Risk of bias of pre-post studies assessed with Methodological Index for Non-randomized Studies (MINORS).

First Author	Year	Clearly stated aim	Consecutive inclusion	Prospective collection of data	Appropriate endpoints	Unbiased assessment of study endpoint	Appropriate follow-up period	Loss to follow up <5%	Prospective calculation of study size	SUM	Overall quality[a]
Jie Wang	2017	7	4	7	5	3	5	3	4	38	High
Ping Wu	2017	5	3	5	4	2	4	4	1	28	Low
Qiujing Jiang	2016	7	5	6	4	3	4	4	2	35	Medium
Junsheng Huo	2015	7	5	7	6	4	6	5	4	44	High
Qiannan Zhang	2015	7	4	7	6	3	4	5	7	43	High
Jianhong Qin	2014	6	3	7	3	2	4	4	2	31	Medium
Zuyang Liu	2013	6	3	6	5	2	5	4	2	33	Medium
Zengkang Xu	2012	7	5	7	5	4	6	4	3	41	High
Caixia Dong	2012	7	4	7	6	3	3	4	4	38	High
Lixiang Li	2012	5	3	6	4	3	2	3	2	28	Low
Lijuan Wang	2011	7	3	7	6	3	2	4	2	34	Medium
Jing Sun	2011	5	3	6	5	4	2	3	2	30	Low
Hong Shen	2011	4	2	5	3	2	2	2	1	21	Low

The overall quality is based on total MINORS score. In pre-post studies, papers with scores higher than 35 were of high quality, 31-35 were of moderate quality, and less than 31 were of low quality.

Table S3: Composition of three types of YYB.

	A<br>(one sachet per day)	B<br>(one sachet per day)	C<br>(one sachet per 10 days)
Protein	3 g	3.8 g	17.1 g
Fat	1 g		10 g
Carbohydrates	6 g		63.1 g
Iron	7.5 mg	6.0 mg	6 mg
Zinc	5 mg	4.1 mg	
Calcium	200 mg	385 mg	523 mg
Vitamin A	250 μg		333 μg
Vitamin B1	0.5 mg		0.54 mg
Vitamin B2	0.5 mg	0.2 mg	0.87 mg
Vitamin B3			1.72 mg
Vitamin B12	0.5 μg		
Vitamin D	5 μg	7 μg	2 mg
Folic acid	75 μg		
Phosphorus			325 mg
**Number of controlled studies**	**11**	**6**	**1**
**Number of pre-post studies**	**14**	**0**	**0**

Table S4: Effects of YYB by intervention duration on hemoglobin concentration (g/L), height (cm), weight (kg) and Z-scores.

**Post-only with concurrent control studies**	Number of studies	Mean difference (95%CI)	P-value for summary effects	I^2 for heterogeneity (%)
Hemoglobin concentration (g/L)				
>12 months	2	6.17 (-0.09, 12.42)	0.053	92
≤12 months	5	3.78 (-0.57, 8.12)	0.088	97
Height (cm)				
>12 months	2	1.10 (-0.77, 2.96)	0.249	77
≤12 months	4	3.16 (1.41, 4.90)	<0.001	93
Weight (kg)				
>12 months	2	0.64 (-0.09, 1.38)	0.085	88
≤12 months	4	0.85 (0.12, 1.59)	0.023	98
Height-for-age Z score (SD)				
>12 months	1	-0.57 (-0.97, -0.17)	0.006	\
≤12 months	2	0.22 (-0.50, 0.95)	0.548	96
Weight-for-height Z score (SD)				
>12 months	1	0.58 (0.24, 0.92)	0.001	\
≤12 months	2	0.19 (0.09, 0.30)	<0.001	22
Weight-for-age Z score (SD)				
>12 months	1	0.54 (0.20, 0.88)	0.002	\
≤12 months	2	0.07 (-0.26, 0.41)	0.669	91
**Pre-post studies**	Number of studies	Mean difference (95%CI)	P-value for summary effects	I^2 for heterogeneity (%)
Hemoglobin concentration (g/L)				

>12 months	4	8.82 (2.02, 15.62)	0.011	99
≤12 months	3	3.59 (0.32, 6.86)	0.031	93
Height (cm)				
>12 months	2	3.27 (2.57, 3.97)	<0.001	0
≤12 months	3	2.01 (0.54, 3.49)	0.007	92
Weight (kg)				
>12 months	2	0.85 (0.35, 1.35)	0.001	87
≤12 months	3	0.63 (0.09, 1.17)	0.023	96
Height-for-age Z score (SD)				
>12 months	3	0.35 (0.24, 0.46)	<0.001	9
≤12 months	2	0.09 (-0.02, 0.20)	0.115	0
Weight-for-height Z score (SD)				
>12 months	2	0.31 (0.03, 0.58)	0.028	80
≤12 months	2	0.25 (-0.44, 0.93)	0.478	98
Weight-for-age Z score (SD)				
>12 months	3	0.23 (-0.21, 0.67)	0.304	95
≤12 months	2	0.29 (-0.32, 0.90)	0.352	98

Table S5: Effects of YYB by intervention duration on prevalence of anemia, stunting, underweight and wasting.

**Post-only with concurrent control studies**	Number of studies	Risk ratio (95%CI)	P-value for summary effects	I^2 for heterogeneity (%)	Risk difference (95%CI)	P-value for summary effects	I^2 for heterogeneity (%)
Anemia							
>12 months	3	0.45 (0.27, 0.75)	0.002	87	-0.22 (-0.35, -0.10)	<0.001	93
≤12 months	8	0.55 (0.41, 0.74)	<0.001	85	-0.10 (-0.16, -0.05)	<0.001	89
Underweight							
>12 months	1	0.68 (0.26, 1.83)	0.449	\	-0.04 (-0.13, 0.06)	0.444	\
≤12 months	5	0.50 (0.38, 0.65)	<0.001	34	-0.04 (-0.06, -0.03)	<0.001	0
Stunting							
>12 months	2	0.48 (0.38, 0.60)	<0.001	0	-0.08 (-0.10, -0.05)	<0.001	0
≤12 months	5	0.63 (0.44, 0.92)	0.016	70	-0.05 (-0.08, -0.02)	0.002	52
Wasting							
>12 months	1	0.77 (0.28, 2.11)	0.612	\	-0.02 (-0.11, 0.07)	0.609	\
≤12 months	4	0.44 (0.29, 0.67)	<0.001	29	-0.03 (-0.04, -0.01)	<0.001	63
**Pre-post studies**	Number of studies	Risk ratio (95%CI)	P-value for summary effects	I^2 for heterogeneity (%)	Risk difference (95%CI)	P-value for summary effects	I^2 for heterogeneity (%)
Anemia							
>12 months	8	0.53 (0.45, 0.64)	<0.001	84	-0.24 (-0.32, -0.15)	<0.001	94
≤12 months	5	0.70 (0.58, 0.85)	<0.001	81	-0.14 (-0.21, -0.06)	<0.001	82
Underweight							
>12 months	6	0.64 (0.39, 1.04)	0.071	70	-0.03 (-0.06, 0.00)	0.083	81

≤12 months	4	0.52 (0.30, 0.90)	0.019	61	-0.03 (-0.05, -0.01)	0.004	51
Stunting							
>12 months	6	0.72 (0.51, 1.03)	0.073	77	-0.04 (-0.08, 0.00)	0.069	78
≤12 months	4	0.81 (0.61, 1.07)	0.131	37	-0.02 (-0.05, 0.00)	0.088	38
Wasting							
>12 months	5	0.71 (0.51, 0.98)	0.035	0	-0.01 (-0.03, 0.00)	0.126	38
≤12 months	2	0.51 (0.05, 5.29)	0.569	89	-0.01 (-0.07, 0.05)	0.741	92

Table S6: Effects of YYB on hemoglobin concentration (g/L), height (cm), weight (kg) and Z-scores among studies of high and medium quality[1].

**Post-only with concurrent control studies**	Number of studies	Mean difference (95%CI)	P-value for summary effects	I^2 for heterogeneity (%)
Hemoglobin concentration (g/L)	6	5.23 (2.14, 8.31)	0.001	97
Height (cm)	5	2.82 (0.89, 4.75)	0.004	95
Weight (kg)	5	0.85 (0.19, 1.51)	0.011	97
Height-for-age Z score (SD)	3	-0.03 (-0.68, 0.62)	0.938	95
Weight-for-height Z score (SD)	3	0.27 (0.09, 0.45)	0.003	66
Weight-for-age Z score (SD)	3	0.20 (-0.11, 0.51)	0.213	89
**Pre-post studies**	Number of studies	Mean difference (95%CI)	P-value for summary effects	I^2 for heterogeneity (%)
Hemoglobin concentration (g/L)	6	6.44 (2.21, 10.67)	0.003	99
Height (cm)	4	2.26 (0.99, 3.52)	<0.001	90
Weight (kg)	4	0.59 (0.23, 0.96)	0.001	92
Height-for-age Z score (SD)	4	0.28 (0.11, 0.45)	0.001	73
Weight-for-height Z score (SD)	3	0.17 (-0.15, 0.49)	0.304	92
Weight-for-age Z score (SD)	4	0.17 (-0.15, 0.48)	0.301	94

[1] High and medium quality studies refers to post-only with concurrent control studies with total quality scores ≥50, and pre-post studies with total quality scores ≥31.

Table S7: Effects of YYB on prevalence of anemia, stunting, underweight and wasting among studies of high and medium quality[1].

**Post-only with concurrent control studies**	Number of studies	Risk ratio (95%CI)	P-value for summary effects	I^2 for heterogeneity (%)	Risk difference (95%CI)	P-value for summary effects	I^2 for heterogeneity (%)
Anemia	9	0.53 (0.44, 0.64)	<0.001	75	-0.15 (-0.23, -0.07)	<0.001	96
Underweight	4	0.42 (0.29, 0.59)	<0.001	0	-0.04 (-0.05, -0.02)	<0.001	0
Stunting	5	0.54 (0.38, 0.78)	0.001	76	-0.06 (-0.09, -0.03)	<0.001	62
Wasting	4	0.45 (0.30, 0.68)	<0.001	29	-0.03 (-0.05, -0.00)	0.026	57
**Pre-post studies**	Number of studies	Risk ratio (95%CI)	P-value for summary effects	I^2 for heterogeneity (%)	Risk difference (95%CI)	P-value for summary effects	I^2 for heterogeneity (%)
Anemia	9	0.61 (0.51, 0.73)	<0.001	91	-0.21 (-0.29, -0.13)	<0.001	95
Underweight	8	0.69 (0.51, 0.93)	0.017	50	-0.02 (-0.04, -0.00)	0.026	60
Stunting	8	0.79 (0.62, 1.01)	0.057	65	-0.03 (-0.05, -0.00)	0.041	64
Wasting	5	0.93 (0.70, 1.23)	0.599	40	-0.00 (-0.01, 0.01)	0.595	42

[1] High and medium quality studies refer to post-only with concurrent control studies with total quality scores ≥50, and pre-post studies with total quality scores ≥31.

(a)

Study or Subgroup	Intervention Mean	Intervention SD	Intervention Total	Control Mean	Control SD	Control Total	Weight	Mean Difference IV, Random, 95% CI
Lingyun Ren 2014	118.5	10.8	76	108.9	11	78	12.5%	9.60 [6.16, 13.04]
Qin Hu 2016	114.3	4.3	300	111.1	4.3	300	15.1%	3.20 [2.51, 3.89]
Shuai Li 2017	118.5	9.6	450	107.2	10.7	450	14.7%	11.30 [9.97, 12.63]
Songli Fan 2013	110.14	9.76	328	110.58	9.89	113	14.1%	-0.44 [-2.55, 1.67]
Xiaoting Ding 2016	117.4	12.1	483	113.1	12.2	248	14.3%	4.30 [2.44, 6.16]
Yuying Wang 2004	128	9.6	670	126.2	10.2	307	14.7%	1.80 [0.45, 3.15]
Yuying Wang 2009	127.8	9.7	503	126	10	218	14.6%	1.80 [0.22, 3.38]
**Total (95% CI)**			**2810**			**1714**	**100.0%**	**4.43 [1.55, 7.30]**

Heterogeneity: Tau² = 14.13; Chi² = 166.20, df = 6 (P < 0.00001); I² = 96%

Test for overall effect: Z = 3.02 (P = 0.003)

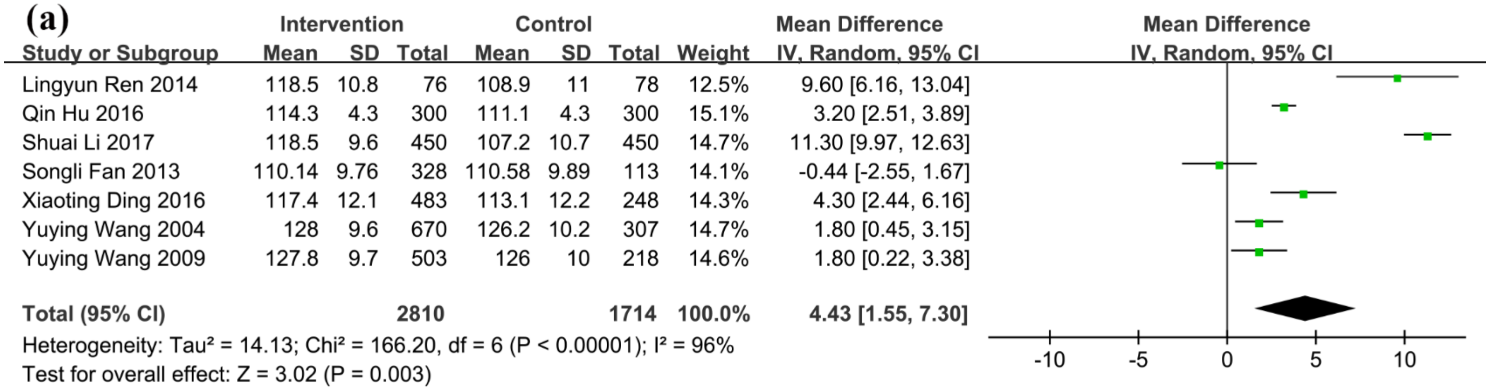

(b)

Study or Subgroup	Posttest Mean	Posttest SD	Posttest Total	Pretest Mean	Pretest SD	Pretest Total	Weight	Mean Difference IV, Random, 95% CI
Caixia Dong 2012	116.76	11.81	242	106.18	12.72	314	14.0%	10.58 [8.53, 12.63]
Junsheng Huo 2015	117	12	1040	108	13	1290	14.5%	9.00 [7.98, 10.02]
Lijuan Wang 2011	120.8	11.2	253	105.7	11.6	257	14.1%	15.10 [13.12, 17.08]
Lixiang Li 2012	116.8	11.4	307	109.4	12.3	327	14.1%	7.40 [5.56, 9.24]
Qiannan Zhang 2015	117.78	11.12	589	116.42	12.39	596	14.4%	1.36 [0.02, 2.70]
Qiujing Jiang 2016	110.4	12.5	639	108.2	12.5	706	14.4%	2.20 [0.86, 3.54]
Zengkang Xu 2012	11.7	1.15	300	10.94	1.23	327	14.6%	0.76 [0.57, 0.95]
**Total (95% CI)**			**3370**			**3817**	**100.0%**	**6.58 [2.71, 10.45]**

Heterogeneity: Tau² = 26.68; Chi² = 561.75, df = 6 (P < 0.00001); I² = 99%

Test for overall effect: Z = 3.33 (P = 0.0009)

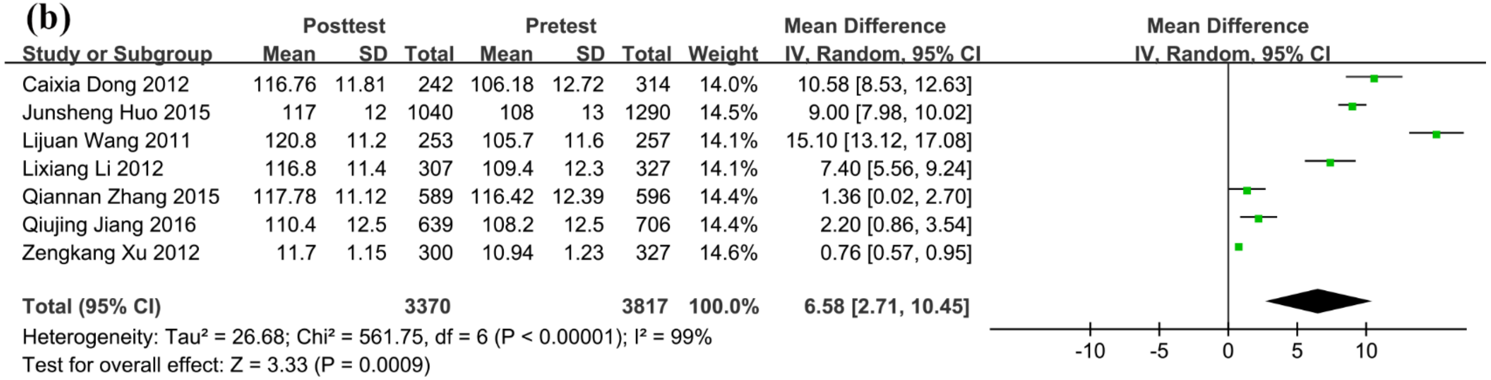

Figure S1: Effects of YYB on hemoglobin level (g/L) among post-only with concurrent control studies (a) and pre-post studies (b) using mean difference as the effect measure.

(a)

Study or Subgroup	Intervention Mean	Intervention SD	Intervention Total	Control Mean	Control SD	Control Total	Weight	Mean Difference IV, Random, 95% CI
Lingyun Ren 2014	78.06	5.64	76	75.83	5.1	78	16.1%	2.23 [0.53, 3.93]
Qin Hu 2016	89.2	4	300	88.9	3.6	300	19.6%	0.30 [-0.31, 0.91]
Shuai Li 2017	89.48	6.41	450	85.76	5.48	450	19.2%	3.72 [2.94, 4.50]
Shuhua Ni 1995	74.78	2.11	76	73.6	1.65	77	19.6%	1.18 [0.58, 1.78]
Wenli Zhao 2012	99.8	51.7	676	94.2	38.3	536	6.1%	5.60 [0.53, 10.67]
Xiaoting Ding 2016	82.9	4.2	483	79	4.7	248	19.4%	3.90 [3.21, 4.59]
**Total (95% CI)**			**2061**			**1689**	**100.0%**	**2.46 [0.96, 3.97]**

Heterogeneity: Tau² = 2.92; Chi² = 86.56, df = 5 (P < 0.00001); I² = 94%
Test for overall effect: Z = 3.20 (P = 0.001)

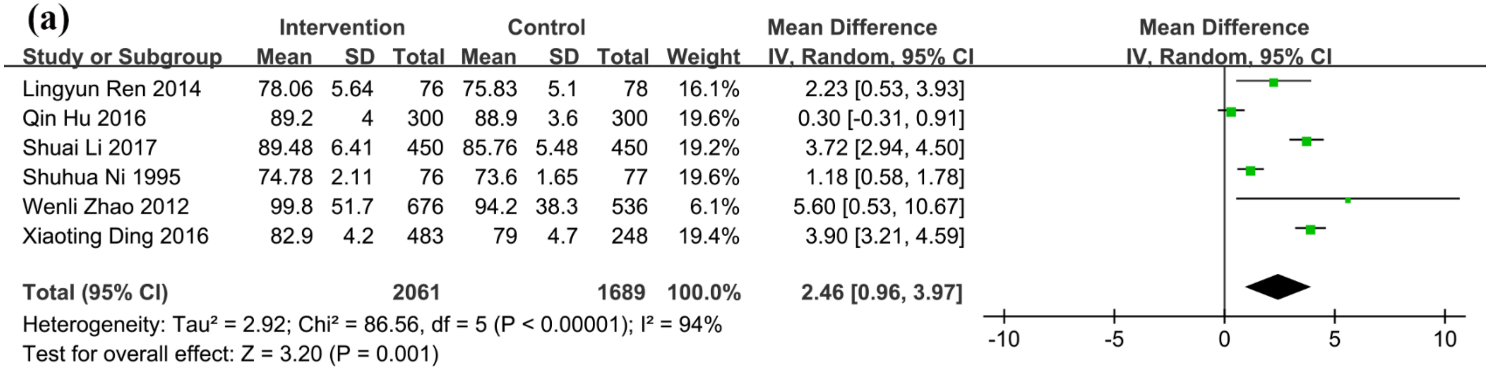

(b)

Study or Subgroup	Posttest Mean	Posttest SD	Posttest Total	Pretest Mean	Pretest SD	Pretest Total	Weight	Mean Difference IV, Random, 95% CI
Lijuan Wang 2011	79.6	7.1	253	76.6	6.8	257	17.9%	3.00 [1.79, 4.21]
Lixiang Li 2012	77.8	5.1	307	74.5	5.5	327	20.2%	3.30 [2.47, 4.13]
Qiannan Zhang 2015	77.97	5.94	589	75.85	6.12	596	20.9%	2.12 [1.43, 2.81]
Qiujing Jiang 2016	75.19	5.94	639	74.52	5.86	706	21.1%	0.67 [0.04, 1.30]
Zengkang Xu 2012	77.9	5.48	300	74.5	5.5	327	20.0%	3.40 [2.54, 4.26]
**Total (95% CI)**			**2088**			**2213**	**100.0%**	**2.46 [1.35, 3.58]**

Heterogeneity: Tau² = 1.44; Chi² = 38.56, df = 4 (P < 0.00001); I² = 90%
Test for overall effect: Z = 4.32 (P < 0.0001)

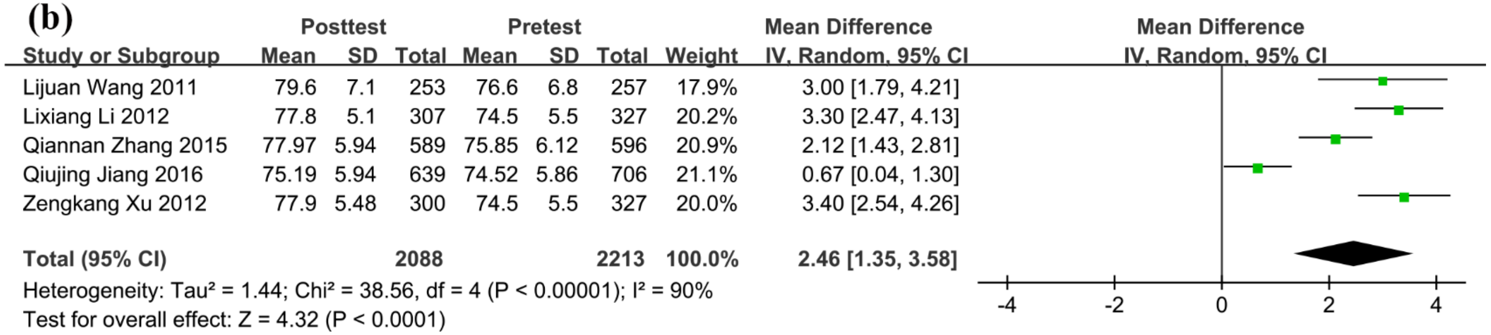

Figure S2: Effects of YYB on height (cm) among post-only with concurrent control studies (a) and pre-post studies (b) using mean difference as the effect measure.

(a)

Study or Subgroup	Intervention Mean	Intervention SD	Intervention Total	Control Mean	Control SD	Control Total	Weight	Mean Difference IV, Random, 95% CI
Lingyun Ren 2014	10.45	1.56	76	9.4	1.45	78	15.4%	1.05 [0.57, 1.53]
Qin Hu 2016	11.6	1.3	300	11.3	1.2	300	17.1%	0.30 [0.10, 0.50]
Shuai Li 2017	13.17	1.57	450	11.45	1.32	450	17.2%	1.72 [1.53, 1.91]
Shuhua Ni 1995	9.41	0.6	76	8.92	0.8	77	17.0%	0.49 [0.27, 0.71]
Wenli Zhao 2012	14.9	3.2	676	14	3.8	536	16.0%	0.90 [0.50, 1.30]
Xiaoting Ding 2016	10.1	1.2	483	9.8	1.2	248	17.2%	0.30 [0.12, 0.48]
**Total (95% CI)**			**2061**			**1689**	**100.0%**	**0.79 [0.25, 1.32]**

Heterogeneity: Tau² = 0.43; Chi² = 150.32, df = 5 (P < 0.00001); I² = 97%
Test for overall effect: Z = 2.88 (P = 0.004)

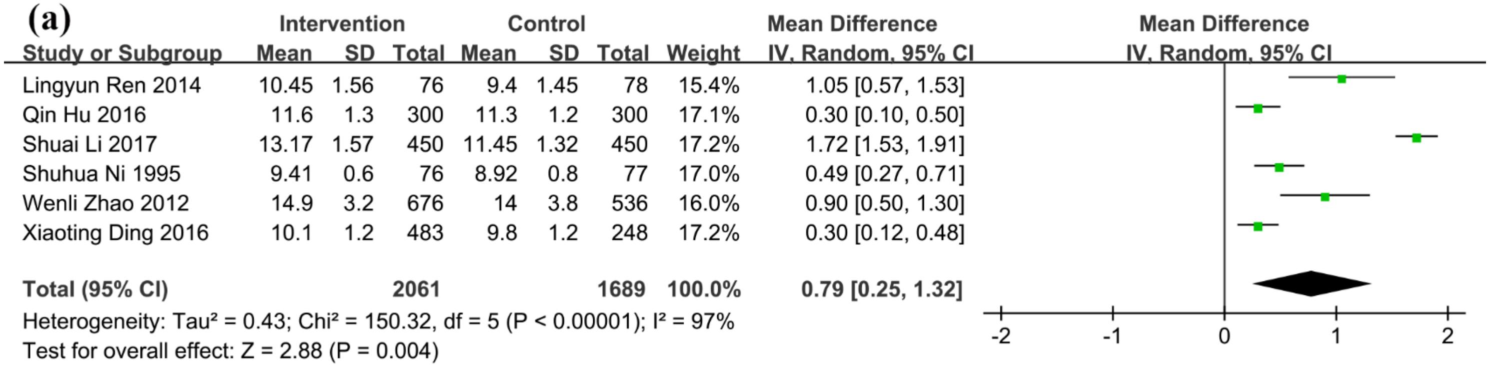

(b)

Study or Subgroup	Posttest Mean	Posttest SD	Posttest Total	Pretest Mean	Pretest SD	Pretest Total	Weight	Mean Difference IV, Random, 95% CI
Lijuan Wang 2011	9.95	1.73	253	9.36	1.57	257	19.1%	0.59 [0.30, 0.88]
Lixiang Li 2012	10.3	1.4	307	9.1	1.3	327	20.1%	1.20 [0.99, 1.41]
Qiannan Zhang 2015	9.79	1.57	301	9.43	1.57	595	20.0%	0.36 [0.14, 0.58]
Qiujing Jiang 2016	9.74	1.48	639	9.41	1.44	706	20.7%	0.33 [0.17, 0.49]
Zengkang Xu 2012	10.22	1.44	300	9.12	1.33	327	20.0%	1.10 [0.88, 1.32]
**Total (95% CI)**			**1800**			**2212**	**100.0%**	**0.72 [0.33, 1.10]**

Heterogeneity: Tau² = 0.18; Chi² = 65.57, df = 4 (P < 0.00001); I² = 94%
Test for overall effect: Z = 3.67 (P = 0.0002)

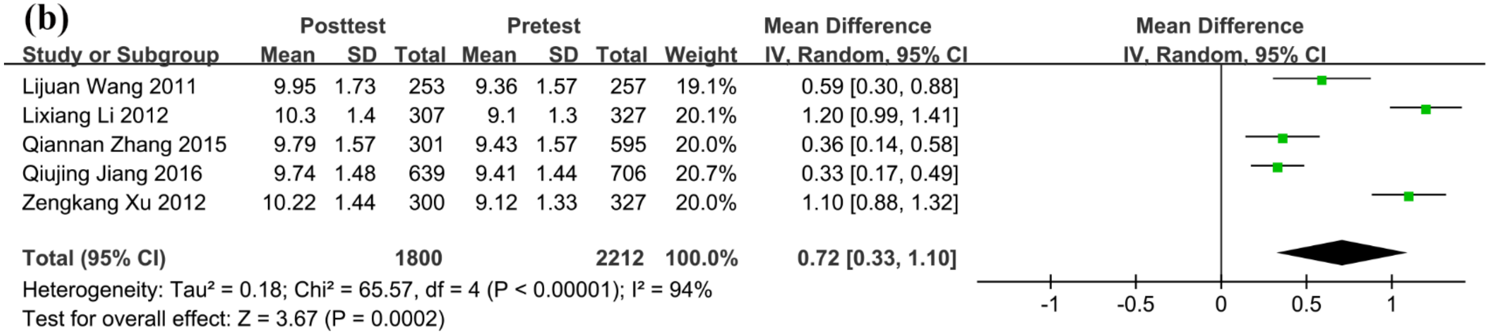

Figure S3: Effects of YYB on weight (kg) among post-only with concurrent control studies (a) and pre-post studies (b) using mean difference as the effect measure.

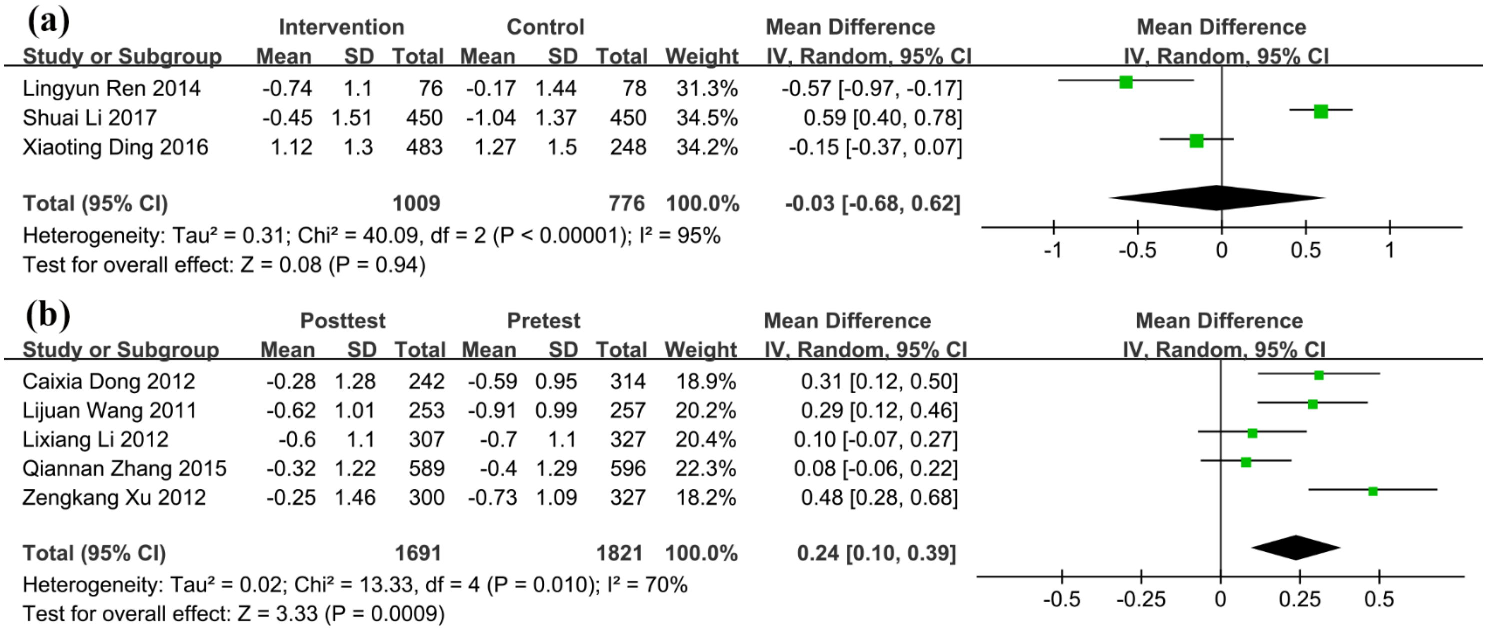

Figure S4: Effects of YYB on height-for-age z score (HAZ) among post-only with concurrent control studies (a) and pre-post studies (b) using mean difference as the effect measure.

(a)

Study or Subgroup	Intervention Mean	Intervention SD	Intervention Total	Control Mean	Control SD	Control Total	Weight	Mean Difference IV, Random, 95% CI
Lingyun Ren 2014	0.42	1.08	76	-0.16	1.08	78	18.0%	0.58 [0.24, 0.92]
Shuai Li 2017	0.68	1.17	450	0.42	1.16	450	38.4%	0.26 [0.11, 0.41]
Xiaoting Ding 2016	0.68	0.89	483	0.53	0.67	248	43.7%	0.15 [0.03, 0.27]
**Total (95% CI)**			**1009**			**776**	**100.0%**	**0.27 [0.09, 0.45]**

Heterogeneity: Tau² = 0.02; Chi² = 5.96, df = 2 (P = 0.05); I² = 66%
Test for overall effect: Z = 2.98 (P = 0.003)

Mean Difference IV, Random, 95% CI

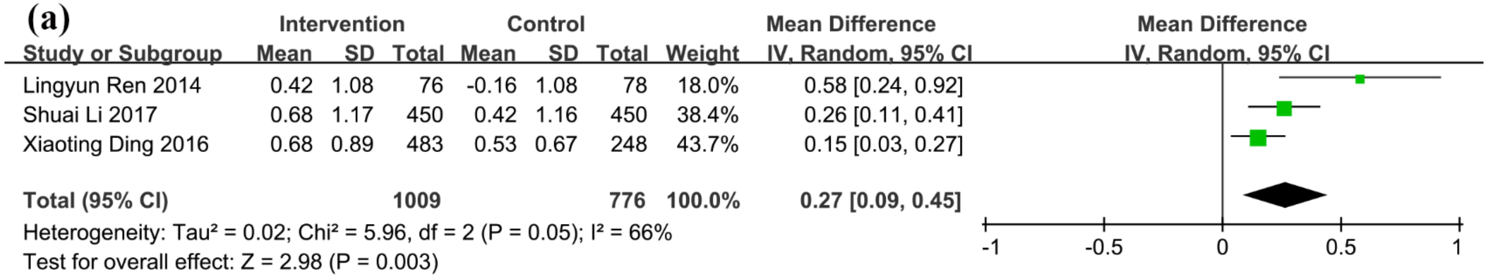

(b)

Study or Subgroup	Posttest Mean	Posttest SD	Posttest Total	Pretest Mean	Pretest SD	Pretest Total	Weight	Mean Difference IV, Random, 95% CI
Caixia Dong 2012	0.13	0.97	242	-0.04	1.06	314	24.8%	0.17 [0.00, 0.34]
Lixiang Li 2012	0.5	0.9	307	-0.1	1.1	327	25.0%	0.60 [0.44, 0.76]
Qiannan Zhang 2015	-0.23	1.09	589	-0.13	1.09	596	25.6%	-0.10 [-0.22, 0.02]
Zengkang Xu 2012	0.3	1.2	300	-0.15	1.09	327	24.6%	0.45 [0.27, 0.63]
**Total (95% CI)**			**1438**			**1564**	**100.0%**	**0.28 [-0.05, 0.61]**

Heterogeneity: Tau² = 0.11; Chi² = 54.92, df = 3 (P < 0.00001); I² = 95%
Test for overall effect: Z = 1.64 (P = 0.10)

Mean Difference IV, Random, 95% CI

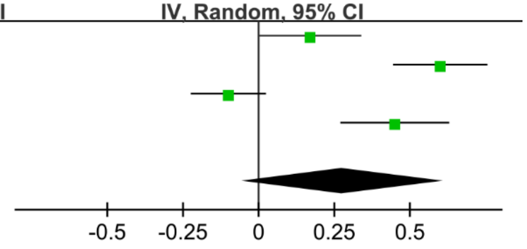

Figure S5: Effects of YYB on weight-for-height z score (WHZ) among post-only with concurrent control studies (a) and pre-post studies (b) using mean difference as the effect measure.

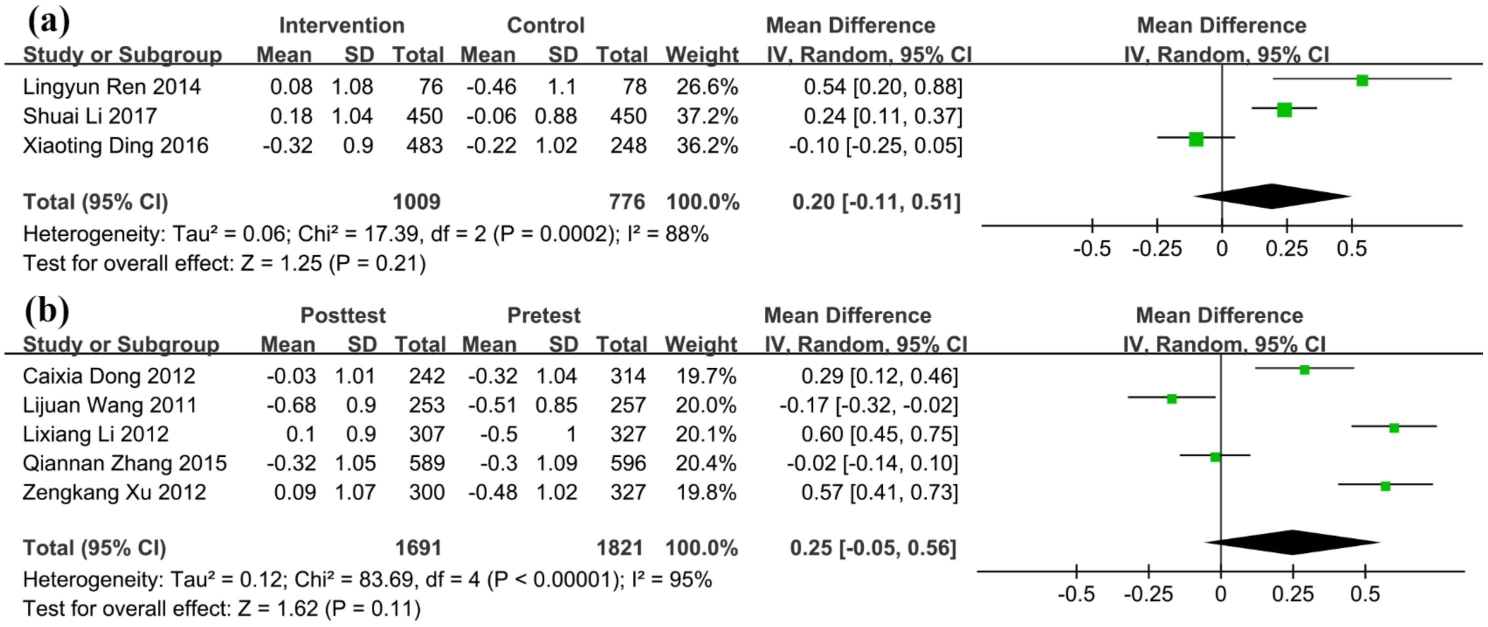

Figure S6: Effects of YYB on weight-for-age z score (WAZ) among post-only with concurrent control studies (a) and pre-post studies (b) using mean difference as the effect measure.

(a)

Study or Subgroup	Intervention Events	Intervention Total	Control Events	Control Total	Weight	Risk Ratio M-H, Random, 95% CI
Lingyun Ren 2014	30	76	52	78	10.6%	0.59 [0.43, 0.82]
Qin Hu 2016	6	300	46	300	4.1%	0.13 [0.06, 0.30]
Shangming Li 2014	179	387	114	240	13.1%	0.97 [0.82, 1.16]
Shuai Li 2017	112	450	226	450	12.9%	0.50 [0.41, 0.60]
Wenhao Li 2013	23	144	46	140	8.5%	0.49 [0.31, 0.76]
Wenli Zhao 2012	9	536	48	676	5.2%	0.24 [0.12, 0.48]
Xiaoting Ding 2016	120	483	98	248	12.4%	0.63 [0.51, 0.78]
Yanfeng Zhang 2016	1039	2174	568	754	14.3%	0.63 [0.60, 0.67]
Yuying Wang 2004	51	670	37	307	9.1%	0.63 [0.42, 0.94]
Yuying Wang 2009	41	503	27	218	8.2%	0.66 [0.42, 1.04]
Zhifeng Fang 2010	2	146	13	107	1.6%	0.11 [0.03, 0.49]
**Total (95% CI)**		**5869**		**3518**	**100.0%**	**0.55 [0.45, 0.67]**
Total events	1612		1275			

Heterogeneity: Tau² = 0.07; Chi² = 61.75, df = 10 (P < 0.00001); I² = 84%
Test for overall effect: Z = 5.92 (P < 0.00001)

(b)

Study or Subgroup	Posttest Events	Posttest Total	Pretest Events	Pretest Total	Weight	Risk Ratio M-H, Random, 95% CI
Caixia Dong 2012	91	242	233	314	8.5%	0.51 [0.43, 0.60]
Hong Shen 2011	13	148	26	143	3.8%	0.48 [0.26, 0.90]
Jianhong Qin 2014	108	206	137	195	8.6%	0.75 [0.64, 0.87]
Jie Wang 2017	138	693	230	823	8.3%	0.71 [0.59, 0.86]
Jing Sun 2011	64	221	82	226	7.4%	0.80 [0.61, 1.04]
Junsheng Huo 2015	257	1040	657	1290	9.0%	0.49 [0.43, 0.55]
Lijuan Wang 2011	78	253	199	257	8.2%	0.40 [0.33, 0.48]
Lixiang Li 2012	76	307	163	327	7.9%	0.50 [0.40, 0.62]
Ping Wu 2017	13	156	51	156	4.2%	0.25 [0.14, 0.45]
Qiannan Zhang 2015	212	589	238	596	8.7%	0.90 [0.78, 1.04]
Qiujing Jiang 2016	247	639	365	706	8.9%	0.75 [0.66, 0.84]
Zengkang Xu 2012	71	300	162	327	7.8%	0.48 [0.38, 0.60]
Zuyang Liu 2013	149	506	292	659	8.6%	0.66 [0.57, 0.78]
**Total (95% CI)**		**5300**		**6019**	**100.0%**	**0.58 [0.50, 0.68]**
Total events	1517		2835			

Heterogeneity: Tau² = 0.07; Chi² = 106.49, df = 12 (P < 0.00001); I² = 89%
Test for overall effect: Z = 6.73 (P < 0.00001)

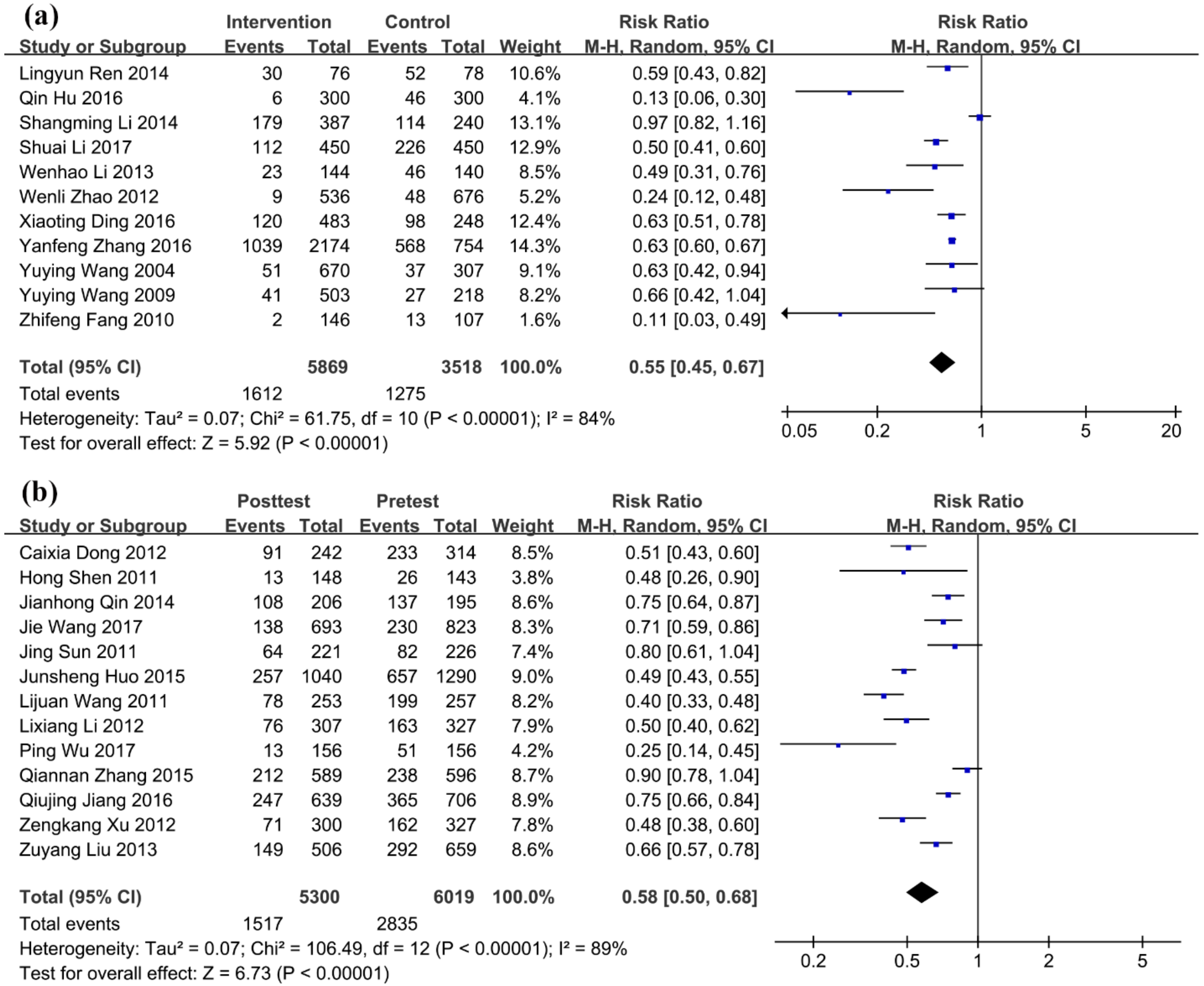

Figure S7: Effects of YYB on anemia prevalence among post-only with concurrent control studies (a) and pre-post studies (b) using risk ratio as the effect measure.

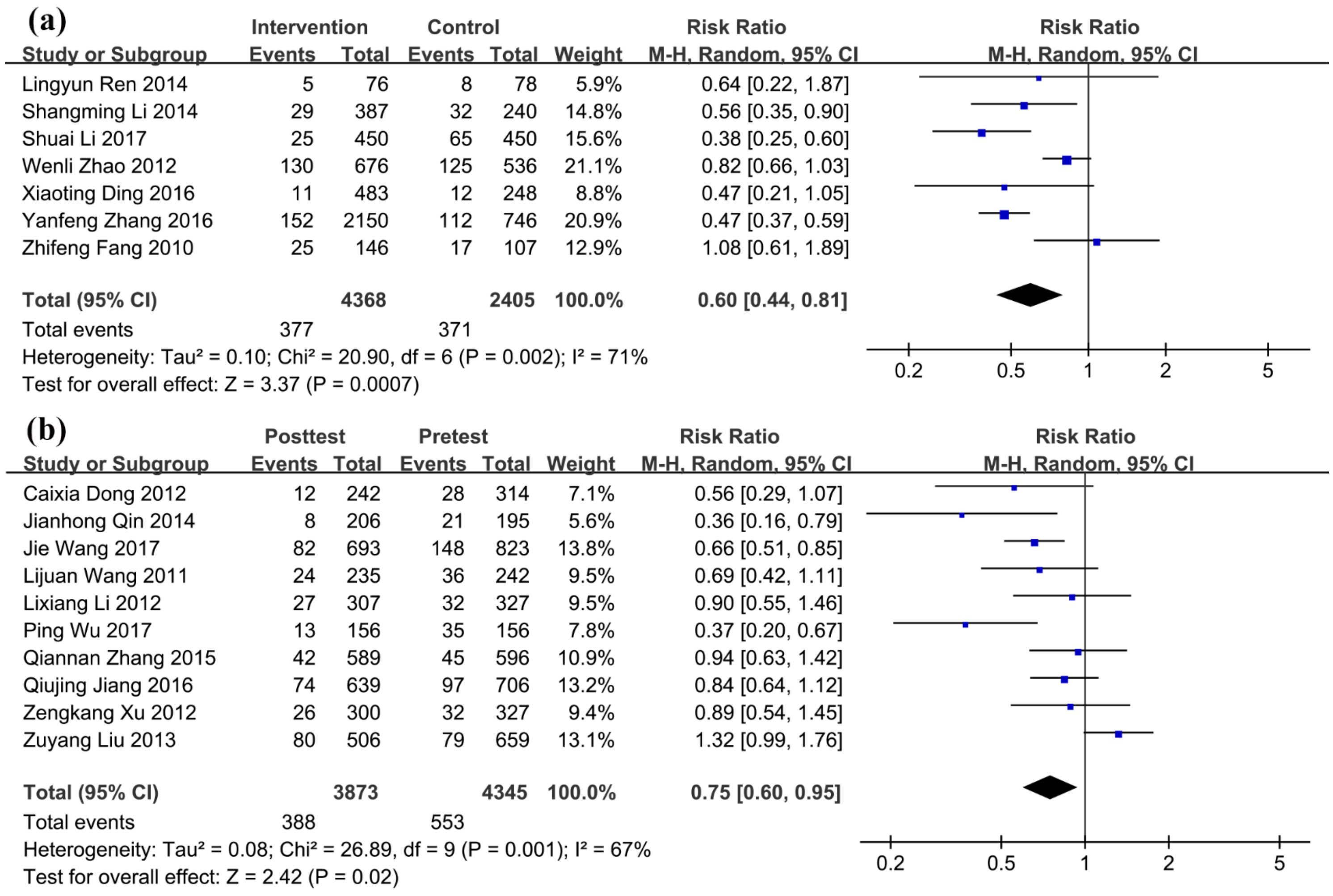

Figure S8: Effects of YYB on stunting prevalence among post-only with concurrent control studies (a) and pre-post studies (b) using risk ratio as the effect measure.

(a)

Study or Subgroup	Intervention Events	Intervention Total	Control Events	Control Total	Weight	Risk Ratio M-H, Fixed, 95% CI
Lingyun Ren 2014	6	76	9	78	5.8%	0.68 [0.26, 1.83]
Shangming Li 2014	29	387	32	240	25.6%	0.56 [0.35, 0.90]
Shuai Li 2017	6	450	26	450	16.9%	0.23 [0.10, 0.56]
Wenli Zhao 2012	19	676	34	536	24.6%	0.44 [0.26, 0.77]
Xiaoting Ding 2016	15	483	16	248	13.7%	0.48 [0.24, 0.96]
Zhifeng Fang 2010	20	146	18	107	13.5%	0.81 [0.45, 1.46]
**Total (95% CI)**		**2218**		**1659**	**100.0%**	**0.51 [0.39, 0.65]**
Total events	95		135			

Heterogeneity: Chi² = 6.39, df = 5 (P = 0.27); I² = 22%
Test for overall effect: Z = 5.22 (P < 0.00001)

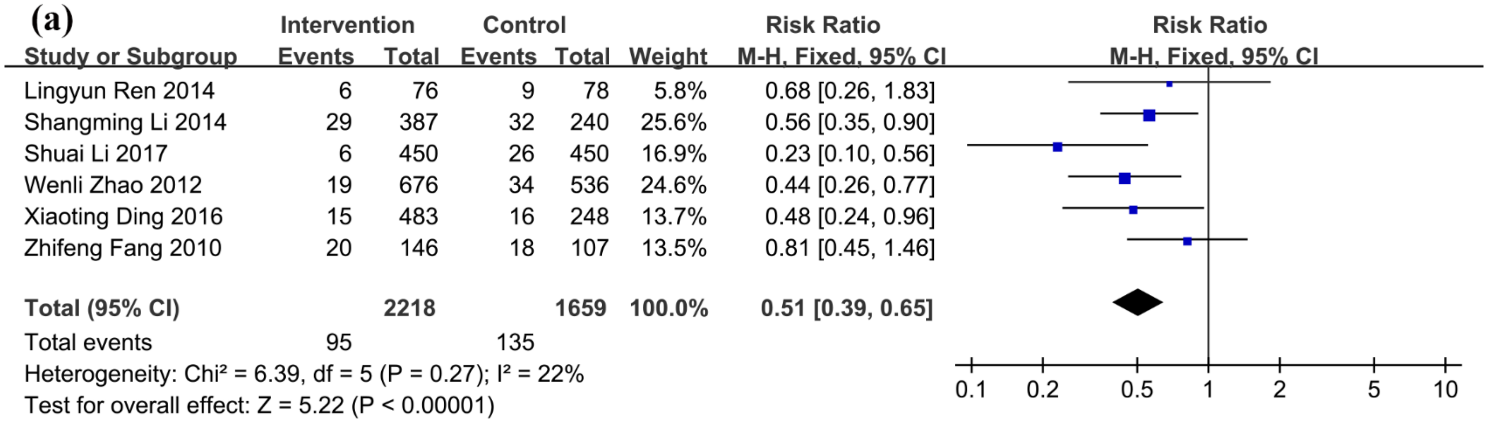

(b)

Study or Subgroup	Posttest Events	Posttest Total	Pretest Events	Pretest Total	Weight	Risk Ratio M-H, Random, 95% CI
Caixia Dong 2012	8	242	14	314	8.2%	0.74 [0.32, 1.74]
Jianhong Qin 2014	14	206	21	195	10.5%	0.63 [0.33, 1.21]
Jie Wang 2017	26	693	77	823	13.2%	0.40 [0.26, 0.62]
Lijuan Wang 2011	14	235	10	242	8.8%	1.44 [0.65, 3.18]
Lixiang Li 2012	3	307	21	327	5.5%	0.15 [0.05, 0.51]
Ping Wu 2017	7	156	27	156	8.7%	0.26 [0.12, 0.58]
Qiannan Zhang 2015	26	589	31	596	12.2%	0.85 [0.51, 1.41]
Qiujing Jiang 2016	14	639	34	706	10.9%	0.45 [0.25, 0.84]
Zengkang Xu 2012	12	300	21	327	10.0%	0.62 [0.31, 1.24]
Zuyang Liu 2013	23	506	29	659	11.9%	1.03 [0.61, 1.76]
**Total (95% CI)**		**3873**		**4345**	**100.0%**	**0.59 [0.42, 0.83]**
Total events	147		285			

Heterogeneity: Tau² = 0.18; Chi² = 24.28, df = 9 (P = 0.004); I² = 63%
Test for overall effect: Z = 3.04 (P = 0.002)

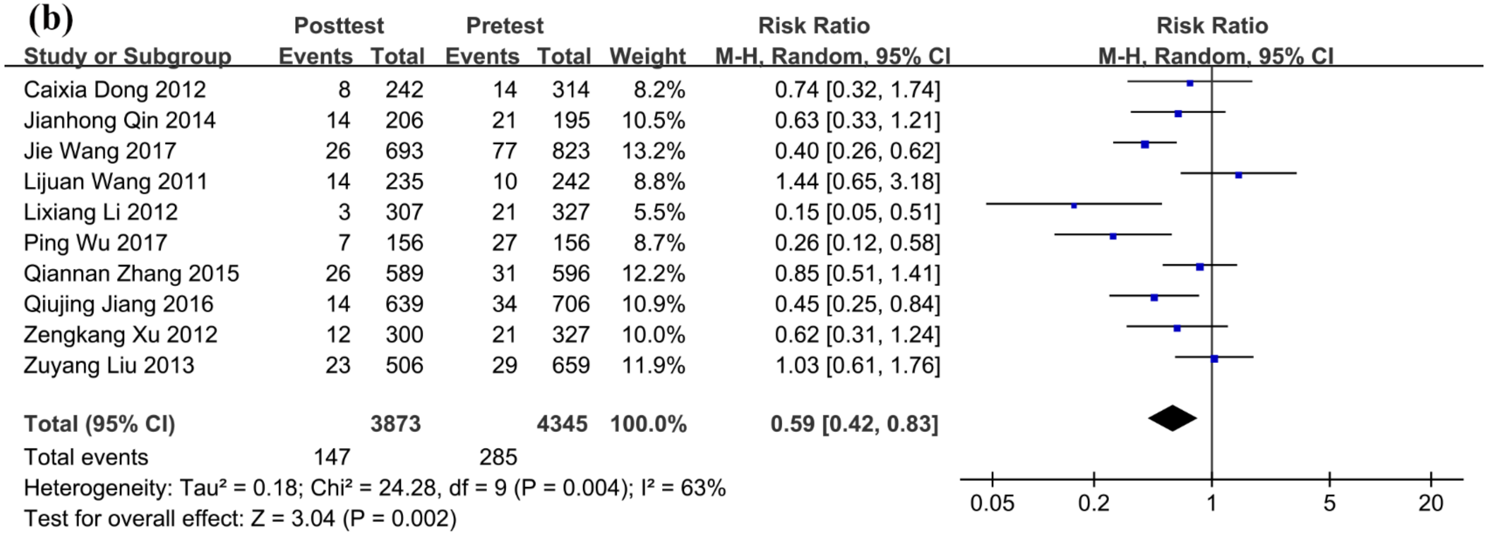

Figure S9: Effects of YYB on underweight prevalence among post-only with concurrent control studies (a) and pre-post studies (b) using risk ratio as the effect measure.

(a)

Study or Subgroup	Intervention Events	Intervention Total	Control Events	Control Total	Weight	Risk Ratio M-H, Fixed, 95% CI
Lingyun Ren 2014	6	76	8	78	10.7%	0.77 [0.28, 2.11]
Shuai Li 2017	8	450	32	450	43.4%	0.25 [0.12, 0.54]
Wenli Zhao 2012	13	676	19	536	28.7%	0.54 [0.27, 1.09]
Xiaoting Ding 2016	9	483	7	248	12.5%	0.66 [0.25, 1.75]
Zhifeng Fang 2010	4	146	3	107	4.7%	0.98 [0.22, 4.28]
**Total (95% CI)**		**1831**		**1419**	**100.0%**	**0.48 [0.32, 0.70]**
Total events	40		69			

Heterogeneity: Chi² = 5.08, df = 4 (P = 0.28); I² = 21%
Test for overall effect: Z = 3.73 (P = 0.0002)

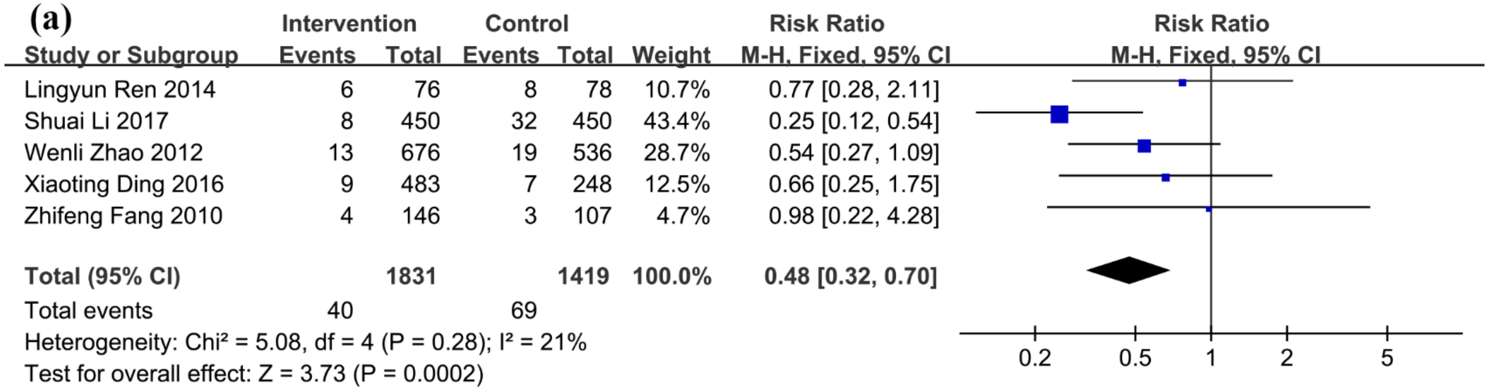

(b)

Study or Subgroup	Posttest Events	Posttest Total	Pretest Events	Pretest Total	Weight	Risk Ratio M-H, Random, 95% CI
Caixia Dong 2012	4	242	11	314	10.3%	0.47 [0.15, 1.46]
Jie Wang 2017	25	693	45	823	20.5%	0.66 [0.41, 1.06]
Lixiang Li 2012	2	307	15	327	7.3%	0.14 [0.03, 0.62]
Ping Wu 2017	6	156	14	156	12.8%	0.43 [0.17, 1.09]
Qiannan Zhang 2015	35	589	24	596	20.0%	1.48 [0.89, 2.45]
Zengkang Xu 2012	14	300	15	327	16.2%	1.02 [0.50, 2.07]
Zuyang Liu 2013	8	506	10	659	12.9%	1.04 [0.41, 2.62]
**Total (95% CI)**		**2793**		**3202**	**100.0%**	**0.72 [0.45, 1.15]**
Total events	94		134			

Heterogeneity: Tau² = 0.22; Chi² = 14.98, df = 6 (P = 0.02); I² = 60%
Test for overall effect: Z = 1.38 (P = 0.17)

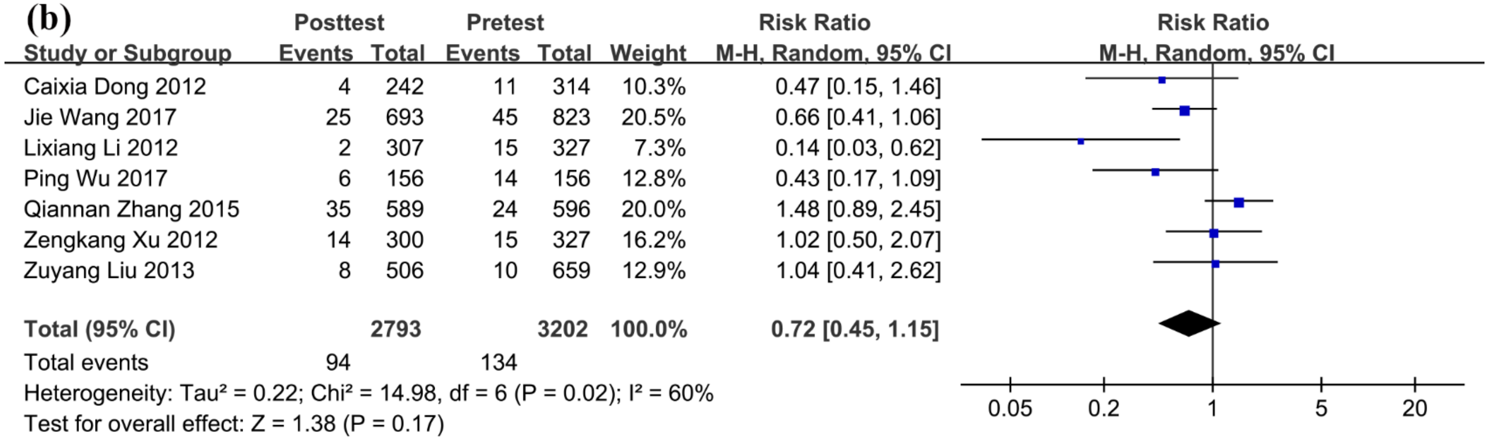

Figure S10: Effects of YYB on wasting prevalence among post-only with concurrent control studies (a) and pre-post studies (b) using risk ratio as the effect measure.

(a)

Study or Subgroup	Intervention Events	Intervention Total	Control Events	Control Total	Weight	Risk Difference M-H, Random, 95% CI
Lingyun Ren 2014	30	76	52	78	6.6%	-0.27 [-0.42, -0.12]
Qin Hu 2016	6	300	46	300	9.7%	-0.13 [-0.18, -0.09]
Shangming Li 2014	179	387	114	240	8.8%	-0.01 [-0.09, 0.07]
Shuai Li 2017	112	450	226	450	9.3%	-0.25 [-0.31, -0.19]
Wenhao Li 2013	23	144	46	140	8.3%	-0.17 [-0.27, -0.07]
Wenli Zhao 2012	9	536	48	676	10.0%	-0.05 [-0.08, -0.03]
Xiaoting Ding 2016	120	483	98	248	9.0%	-0.15 [-0.22, -0.07]
Yanfeng Zhang 2016	1039	2174	568	754	9.8%	-0.28 [-0.31, -0.24]
Yuying Wang 2004	51	670	37	307	9.7%	-0.04 [-0.09, -0.00]
Yuying Wang 2009	41	503	27	218	9.6%	-0.04 [-0.09, 0.01]
Zhifeng Fang 2010	2	146	13	107	9.2%	-0.11 [-0.17, -0.04]
**Total (95% CI)**		**5869**		**3518**	**100.0%**	**-0.13 [-0.20, -0.07]**
Total events	1612		1275			

Heterogeneity: Tau² = 0.01; Chi² = 189.86, df = 10 (P < 0.00001); I² = 95%
Test for overall effect: Z = 3.97 (P < 0.0001)

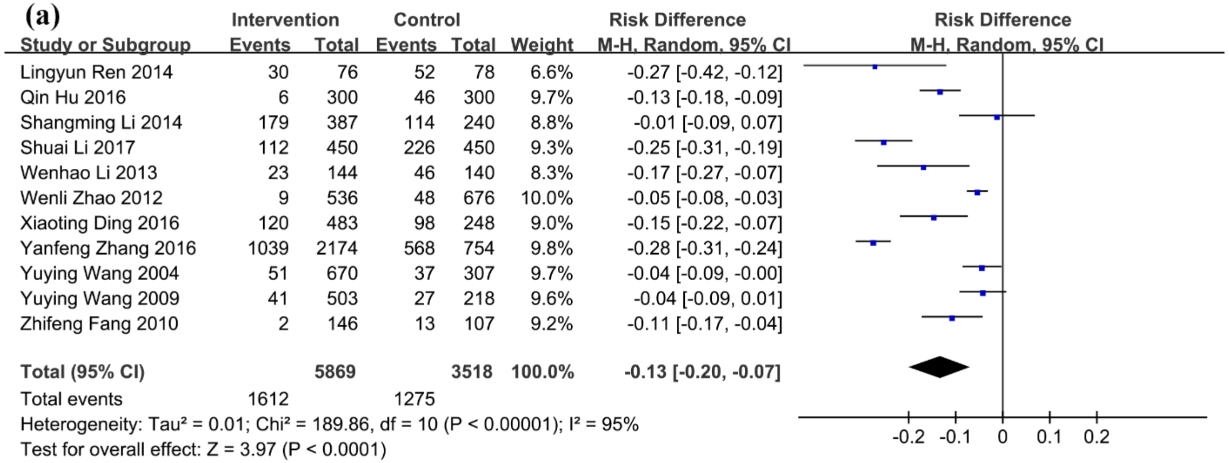

(b)

Study or Subgroup	Posttest Events	Posttest Total	Pretest Events	Pretest Total	Weight	Risk Difference M-H, Random, 95% CI
Caixia Dong 2012	91	242	233	314	7.5%	-0.37 [-0.44, -0.29]
Hong Shen 2011	13	148	26	143	7.5%	-0.09 [-0.17, -0.02]
Jianhong Qin 2014	108	206	137	195	7.2%	-0.18 [-0.27, -0.08]
Jie Wang 2017	138	693	230	823	8.2%	-0.08 [-0.12, -0.04]
Jing Sun 2011	64	221	82	226	7.3%	-0.07 [-0.16, 0.01]
Junsheng Huo 2015	257	1040	657	1290	8.2%	-0.26 [-0.30, -0.22]
Lijuan Wang 2011	78	253	199	257	7.5%	-0.47 [-0.54, -0.39]
Lixiang Li 2012	76	307	163	327	7.6%	-0.25 [-0.32, -0.18]
Ping Wu 2017	13	156	51	156	7.4%	-0.24 [-0.33, -0.16]
Qiannan Zhang 2015	212	589	238	596	8.0%	-0.04 [-0.09, 0.02]
Qiujing Jiang 2016	247	639	365	706	8.0%	-0.13 [-0.18, -0.08]
Zengkang Xu 2012	71	300	162	327	7.6%	-0.26 [-0.33, -0.19]
Zuyang Liu 2013	149	506	292	659	8.0%	-0.15 [-0.20, -0.09]
**Total (95% CI)**		**5300**		**6019**	**100.0%**	**-0.20 [-0.26, -0.13]**
Total events	1517		2835			

Heterogeneity: Tau² = 0.01; Chi² = 164.78, df = 12 (P < 0.00001); I² = 93%
Test for overall effect: Z = 6.07 (P < 0.00001)

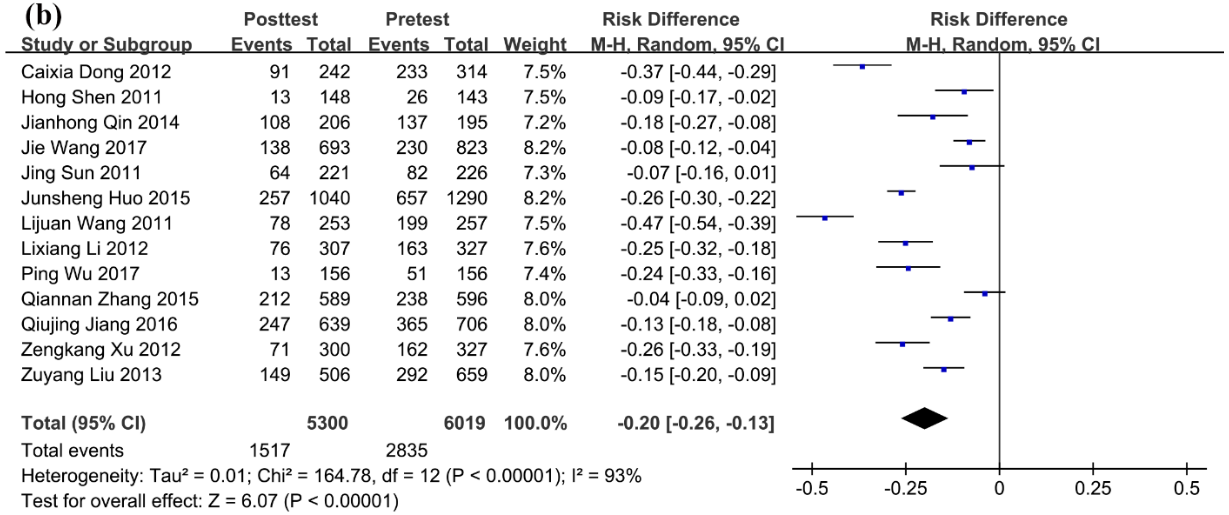

Figure S11: Effects of YYB on anemia prevalence among post-only with concurrent control studies (a) and pre-post studies (b) using risk difference as the effect measure.

(a)

Study or Subgroup	Intervention Events	Intervention Total	Control Events	Control Total	Weight	Risk Difference M-H, Random, 95% CI
Lingyun Ren 2014	5	76	8	78	6.2%	-0.04 [-0.12, 0.05]
Shangming Li 2014	29	387	32	240	13.3%	-0.06 [-0.11, -0.01]
Shuai Li 2017	25	450	65	450	17.3%	-0.09 [-0.13, -0.05]
Wenli Zhao 2012	130	676	125	536	14.5%	-0.04 [-0.09, 0.01]
Xiaoting Ding 2016	11	483	12	248	21.0%	-0.03 [-0.06, 0.00]
Yanfeng Zhang 2016	152	2150	112	746	21.9%	-0.08 [-0.11, -0.05]
Zhifeng Fang 2010	25	146	17	107	5.7%	0.01 [-0.08, 0.10]
**Total (95% CI)**		**4368**		**2405**	**100.0%**	**-0.05 [-0.08, -0.03]**
Total events	377		371			

Heterogeneity: Tau² = 0.00; Chi² = 12.42, df = 6 (P = 0.05); I² = 52%
Test for overall effect: Z = 4.29 (P < 0.0001)

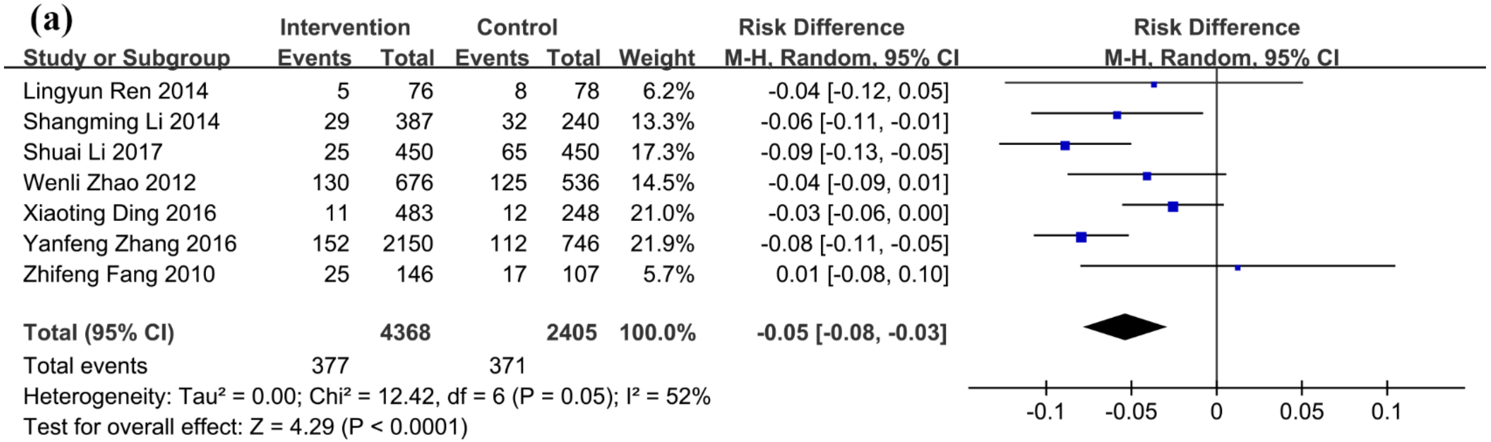

(b)

Study or Subgroup	Posttest Events	Posttest Total	Pretest Events	Pretest Total	Weight	Risk Difference M-H, Random, 95% CI
Caixia Dong 2012	12	242	28	314	10.6%	-0.04 [-0.08, 0.00]
Jianhong Qin 2014	8	206	21	195	9.2%	-0.07 [-0.12, -0.02]
Jie Wang 2017	82	693	148	823	11.5%	-0.06 [-0.10, -0.03]
Lijuan Wang 2011	24	235	36	242	8.0%	-0.05 [-0.11, 0.01]
Lixiang Li 2012	27	307	32	327	10.0%	-0.01 [-0.06, 0.04]
Ping Wu 2017	13	156	35	156	5.9%	-0.14 [-0.22, -0.06]
Qiannan Zhang 2015	42	589	45	596	12.5%	-0.00 [-0.03, 0.03]
Qiujing Jiang 2016	74	639	97	706	11.5%	-0.02 [-0.06, 0.01]
Zengkang Xu 2012	26	300	32	327	10.0%	-0.01 [-0.06, 0.03]
Zuyang Liu 2013	80	506	79	659	10.8%	0.04 [-0.00, 0.08]
**Total (95% CI)**		**3873**		**4345**	**100.0%**	**-0.03 [-0.05, -0.01]**
Total events	388		553			

Heterogeneity: Tau² = 0.00; Chi² = 28.43, df = 9 (P = 0.0008); I² = 68%
Test for overall effect: Z = 2.47 (P = 0.01)

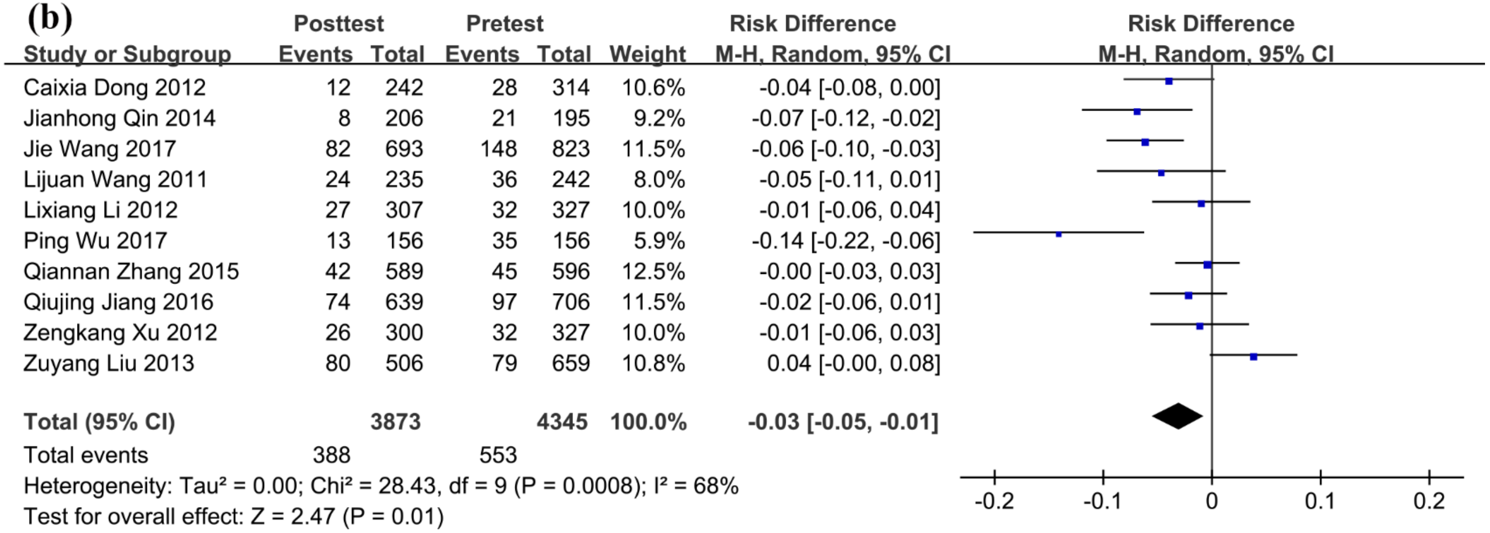

Figure S12: Effects of YYB on stunting prevalence among post-only with concurrent control studies (a) and pre-post studies (b) using risk difference as the effect measure.

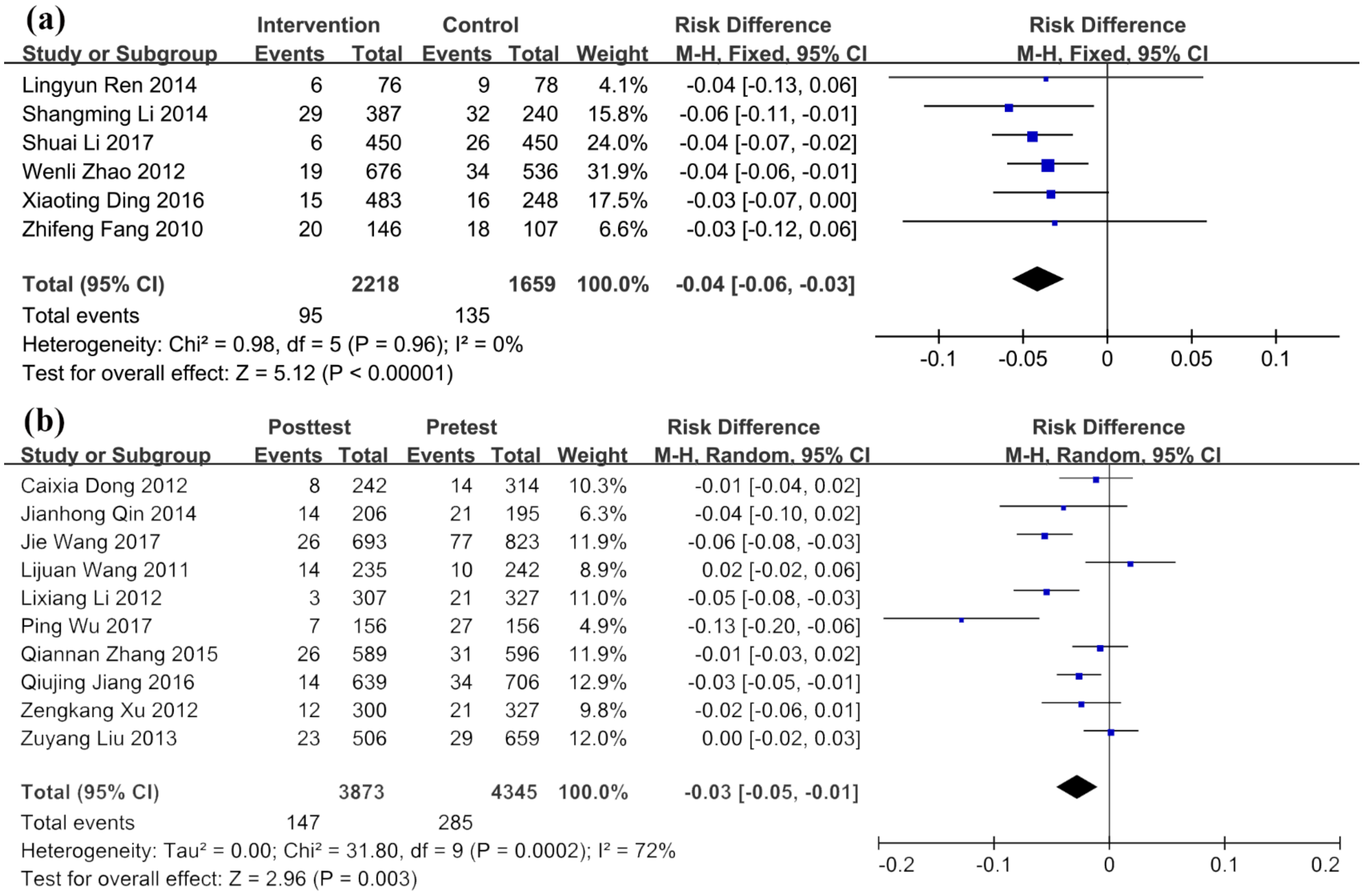

Figure S13: Effects of YYB on underweight prevalence among post-only with concurrent control studies (a) and pre-post studies (b) using risk difference as the effect measure.

(a)

Study or Subgroup	Intervention Events	Intervention Total	Control Events	Control Total	Weight	Risk Difference M-H, Fixed, 95% CI
Lingyun Ren 2014	6	76	8	78	4.9%	-0.02 [-0.11, 0.07]
Shuai Li 2017	8	450	32	450	28.6%	-0.05 [-0.08, -0.03]
Wenli Zhao 2012	13	676	19	536	37.9%	-0.02 [-0.03, 0.00]
Xiaoting Ding 2016	9	483	7	248	20.8%	-0.01 [-0.03, 0.01]
Zhifeng Fang 2010	4	146	3	107	7.8%	-0.00 [-0.04, 0.04]
**Total (95% CI)**		**1831**		**1419**	**100.0%**	**-0.02 [-0.04, -0.01]**
Total events	40		69			

Heterogeneity: Chi² = 8.04, df = 4 (P = 0.09); I² = 50%
Test for overall effect: Z = 3.77 (P = 0.0002)

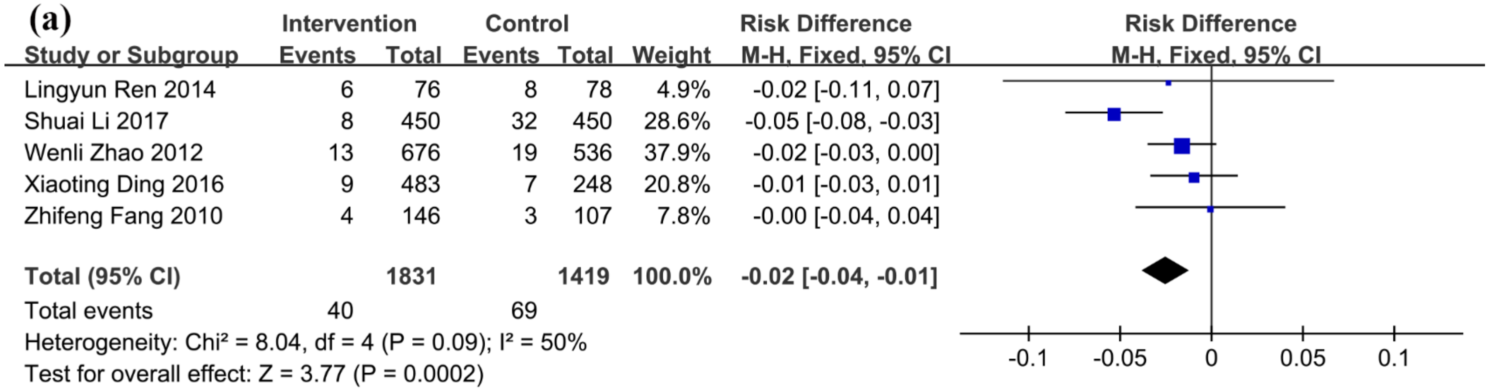

(b)

Study or Subgroup	Posttest Events	Posttest Total	Pretest Events	Pretest Total	Weight	Risk Difference M-H, Random, 95% CI
Caixia Dong 2012	4	242	11	314	14.6%	-0.02 [-0.04, 0.01]
Jie Wang 2017	25	693	45	823	17.0%	-0.02 [-0.04, 0.00]
Lixiang Li 2012	2	307	15	327	15.3%	-0.04 [-0.06, -0.01]
Ping Wu 2017	6	156	14	156	6.3%	-0.05 [-0.11, 0.00]
Qiannan Zhang 2015	35	589	24	596	15.1%	0.02 [-0.01, 0.04]
Zengkang Xu 2012	14	300	15	327	11.7%	0.00 [-0.03, 0.03]
Zuyang Liu 2013	8	506	10	659	20.1%	0.00 [-0.01, 0.01]
**Total (95% CI)**		**2793**		**3202**	**100.0%**	**-0.01 [-0.03, 0.00]**
Total events	94		134			

Heterogeneity: Tau² = 0.00; Chi² = 16.67, df = 6 (P = 0.01); I² = 64%
Test for overall effect: Z = 1.49 (P = 0.14)

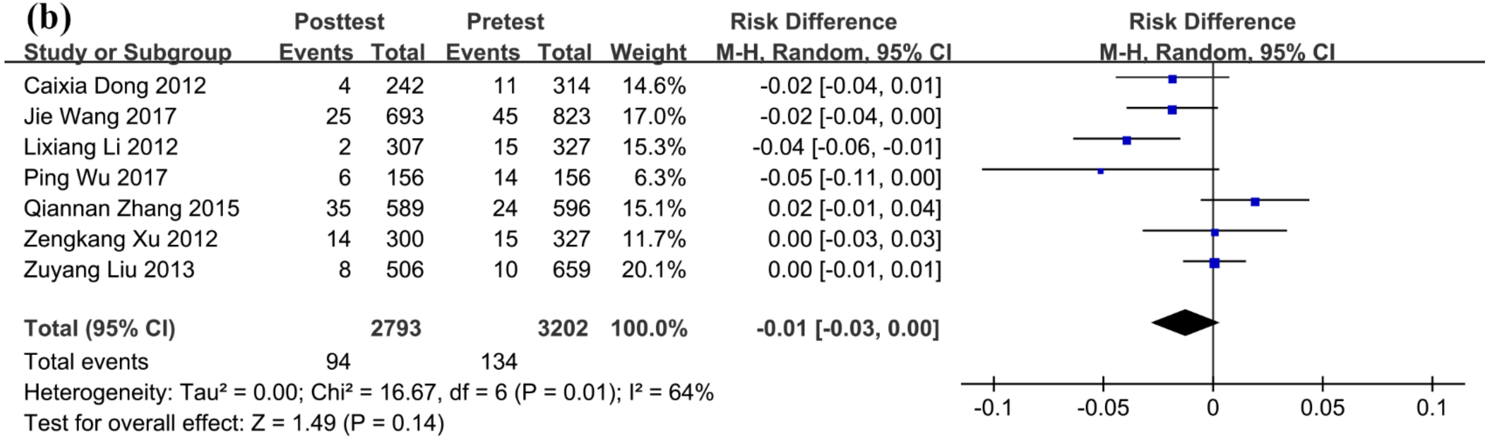

Figure S14: Effects of YYB on wasting prevalence among post-only with concurrent control studies (a) and pre-post studies (b) using risk difference as the effect measure.

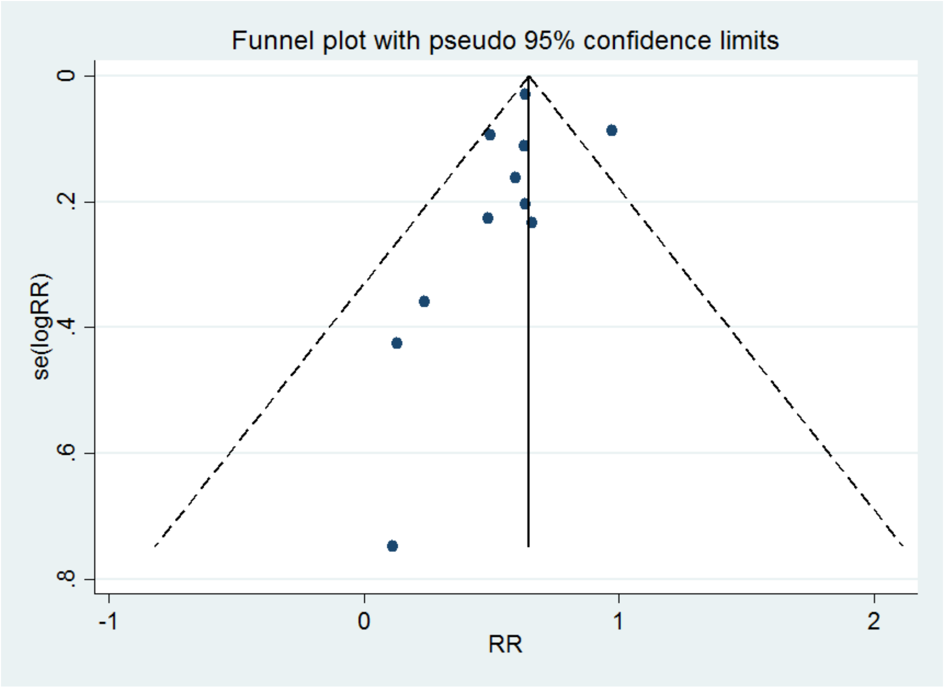

Figure S15: Funnel plot of effects of YYB on anemia prevalence among post-only with concurrent control studies.

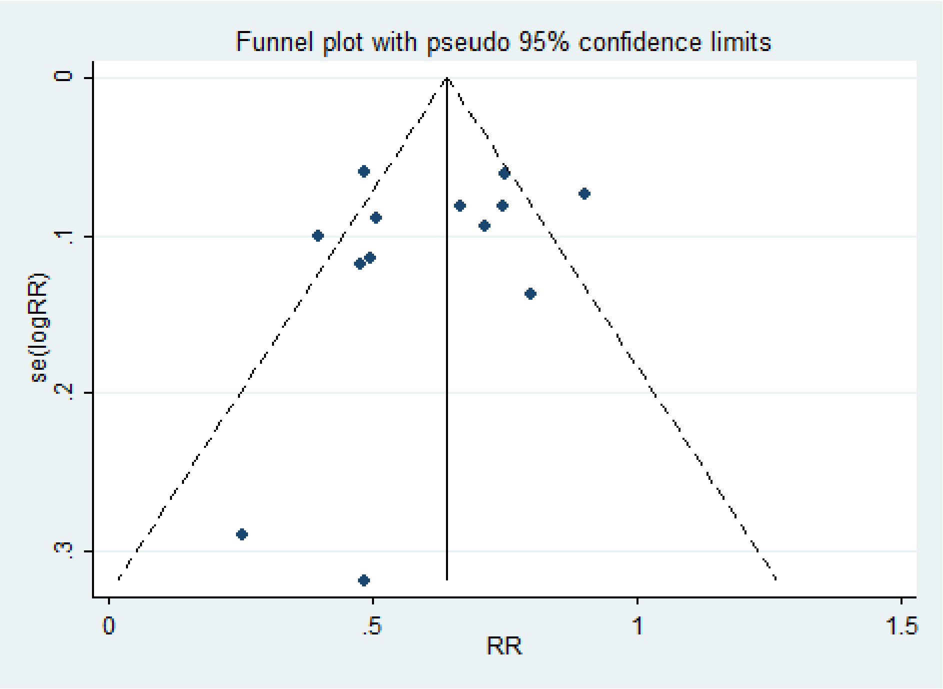

Figure S16: Funnel plot of effects of YYB on anemia prevalence among pre-post studies.
